# Complete Genome Sequence of a Staphylococcus aureus Sequence Type 612 Isolate from an Australian Horse

**DOI:** 10.1128/MRA.00869-18

**Published:** 2018-08-02

**Authors:** Riley J. T. Murphy, Yung Thin Lee, Stanley Pang, Tahlia R. Bastholm, Jade E. Crow, Amy M. Davis, Geoffrey W. Coombs, Mark A. O’Dea, Sam Abraham, Joshua P. Ramsay

**Affiliations:** a Curtin Health Innovation Research Institute and School of Pharmacy and Biomedical Sciences, Curtin University, Perth, Western Australia, Australia; b Antimicrobial Resistance and Infectious Disease (AMRID) Research Laboratory, School of Veterinary and Life Sciences, Murdoch University, Murdoch, Western Australia, Australia; c Department of Microbiology, Fiona Stanley Hospital, PathWest Laboratory Medicine-WA, Murdoch, Western Australia, Australia; University of California, Riverside

## Abstract

Staphylococcus aureus is a serious pathogen of humans and animals. Multilocus sequence type 612 is dominant and highly virulent in South African hospitals but relatively uncommon elsewhere. We present the complete genome sequence of methicillin-resistant Staphylococcus aureus strain SVH7513, isolated from a horse at a veterinary clinic in New South Wales, Australia.

## Abstract (precis)

Staphylococcus aureus is a serious pathogen of humans and animals. Multilocus sequence type 612 is dominant and highly virulent in South African hospitals but relatively uncommon elsewhere.

## ANNOUNCEMENT

Methicillin-resistant Staphylococcus aureus (MRSA) is a serious pathogen of humans and animals. Multilocus sequence type 612 (ST612) is the most commonly isolated sequence type in South African hospital-isolated bacteremia (1, 2). ST612 is relatively uncommon elsewhere but has been sporadically isolated in association with horses and horse veterinarians in Australia (3, 4). ST612 is a close relative of American clones USA500, a health care-associated MRSA strain (5), and the epidemic community-associated USA300 (ST8) (6), both of which are members of clonal complex 8 (CC8). ST612 may pose a threat to public health as a proven human-adapted pathogen and horse-colonizing strain, where colonized horses may act as a reservoir. Here, we present the genome of MRSA strain SVH7513, isolated from a horse at a veterinary clinic in New South Wales, Australia. This genome may present clues to the genetic requirements of equine zoonosis; it provides a quality reference for the assembly of other ST612 genomes and is the first published complete sequence of an ST612 isolate.

SVH7513 was grown in tryptic soy broth (BD Biosciences, Sparks, MD), and cells were lysed in a FavorPrep plasmid extraction minikit (Favorgen, Taiwan) with buffer FAPD 1, containing RNase, with the addition of lysostaphin (Sigma-Aldrich; 2 mg/ml, 37°C, 20 min). Genomic DNA was extracted using a blood genomic DNA extraction kit (Favorgen). The genome of strain SVH7513 was sequenced using Pacific Biosciences (PacBio) RS II single-molecule real-time (SMRT) cell sequencing technology (Macrogen, South Korea). SMRT cell sequencing produced 152,425 subreads with an average subread length of 11,790 bp. Additionally, an Illumina genomic library was prepared using the Nextera XT Library prep, which was sequenced using the Illumina MiSeq platform and 600-cycle reagent kit (v3) (2 × 300-bp paired-end reads), producing 295,192 reads. PacBio reads were assembled with Canu (v1.6) using default parameters, which produced a circular 2,915,384-bp chromosome and a circular 27,887-bp plasmid, pSVH7513a.

The plasmid and chromosome had a 616-fold average depth of coverage. MiSeq reads mapped to these contigs with an average 29-fold depth of coverage. Mapping was used to identify likely sequence errors (Burrows-Wheeler Aligner [BWA] v0.7.17-r1188), but no differences between the Canu assembly and BWA-mapped reads were detected. Separate SPAdes (v3.11.1) assembly, using default parameters, of unmapped Illumina reads (extracted using SAMtools v1.6) identified a 2,495-bp *ermC* plasmid, pSVH7513b.

The assembled sequences were trimmed to remove overlaps, and the origin of the genome was set to *dnaA* using Circlator (v1.5.5). Plasmid origins were manually oriented to the start codon of predicted plasmid replication genes. Annotation was performed using the NCBI Prokaryotic Genome Annotation Pipeline. A total of 3,122 chromosomal genes were identified, of which 3,040 were coding sequences (CDS), 109 were pseudogenes, 19 were rRNA genes, and 59 were tRNA genes.

### Data availability.

This whole-genome sequencing project has been deposited in GenBank under the accession numbers CP029166, CP029167, and CP029165 for the genome and plasmids pSVH7513a and pSVH7513b, respectively.
